# Galectin-8 Contributes to Human Trophoblast Cell Invasion

**DOI:** 10.3390/ijms251810096

**Published:** 2024-09-20

**Authors:** Janko Legner, Milica Jovanović Krivokuća, Aleksandra Vilotić, Andrea Pirković, Mirjana Nacka-Aleksić, Žanka Bojić-Trbojević

**Affiliations:** Department for Biology of Reproduction, Institute for the Application of Nuclear Energy, University of Belgrade, 11000 Belgrade, Serbia; janko.legner@inep.co.rs (J.L.); aleksandrav@inep.co.rs (A.V.); andrea.pirkovic@inep.co.rs (A.P.); mnacka@inep.co.rs (M.N.-A.)

**Keywords:** galectin-8, extravillous trophoblast, invasion, integrins, MMPs

## Abstract

Galectins are a class of lectins that are extensively expressed in all organisms. Galectins are involved in a range of functions, including early development, tissue regeneration, cancer and inflammation. It has been shown that galectin-8 is expressed in the villous and extravillous trophoblast (EVT) cells of the human placenta; however, its physiological role in pregnancy establishment has not been elucidated. Taking these factors into account, we investigated the functional role of galectin-8 in HTR-8/SVneo cells—a human EVT cell line—and human primary cytotrophoblast cells isolated from a first-trimester placenta. We analyzed the effects of recombinant human galectin-8 (rh galectin-8) on the adhesion, migration and invasion of HTR-8/SVneo cells. We used qPCR, cell-based ELISA (cELISA) and gelatin zymography to study the effects of galectin-8 on mediators of these processes, such as integrin subunits alpha-1 and beta-1 and matrix metalloproteinases (MMPs)-2 and -9, on the mRNA and protein levels. Further, we studied the effects of galectin-8 on primary cytotrophoblast cells’ invasion. Galectin-8 stimulated the adhesion, migration and invasion of HTR-8/SVneo cells, as well as the invasion of primary cytotrophoblasts. In addition, the MMP-2 and -9 levels were increased, while the expression of integrins alpha-1 and beta-1 was not affected. Galectin-8 has the ability to positively affect EVTs’ invasion, so it can be considered a significant factor in the trophoblast cell invasion process.

## 1. Introduction

The placenta is a temporary organ that is essential for a fetus to survive. It forms from the blastocyst at the beginning of pregnancy [1] and acts as a bridge between the mother and fetus [2]. The placenta is a hemochorial organ in humans, where placental cells are in contact with maternal blood [3], and its function is, among others, to supply nutrients to the embryo [4]. Trophoblast cells are specific placental cells that differentiate into two main trophoblast subpopulations—syncytiotrophoblasts and extravillous trophoblasts (EVTs) [5]. Syncytiotrophoblasts envelop floating chorionic villi, which are implicated in nutrient and gas exchange between the mother and the fetus [5]. EVTs invade the maternal decidua and myometrium, as well as the maternal vasculature, where they remodel the spiral arteries [6]. Abnormal trophoblast invasion into uterine tissue may lead to pregnancy pathologies such as pre-eclampsia and intrauterine growth restriction (IUGR) [7].

Trophoblast invasion during implantation and placentation is a strongly controlled process [8]. In vitro studies suggest that the various factors responsible for the regulation of this process are autocrine (trophoblast-derived) and paracrine (endometrial) and include cytokines, growth factors, hormones and extracellular matrix (ECM) components [8]. It is well documented that the interaction of cell membrane receptors that mediate cell adhesion—integrins—with the ECM components is of particular importance for trophoblast cell invasion, as well as the expression/activity of MMPs, which are directly or indirectly affected by these regulators [8]. Galectins are the most predominantly expressed class of lectins in all living organisms [9]. They bind β-galactose-containing glycoconjugates and all have a carbohydrate recognition domain (CRD) [9]. Based on the number and organization of their CRDs, galectins have been classified into prototypical, chimera-type and tandem-repeat galectins [9]. These lectins appear in a variety of tissue types and cells and are involved in many diverse biological processes [10], such as early development, tissue regeneration, cancer and immune responses [9]. It has been shown that some galectins have the potential to act as pro-invasive autocrine/paracrine factors in human trophoblast invasion in vitro, such as galectin-1 [11] and galectin-3 [12].

Galectin-8 is a 34 kDa tandem repeat-type galectin, consisting of two CRDs joined by a linker peptide [13]. As mentioned in the review by Bidon et al., there are six different isoforms of galectin-8, resulting from alternative splicing [14]. It was suggested that galectin-8 functions in a fine balancing act between its effects on the expression of cytokines/chemokines, which promote cancer growth, and cytokine-mediated immune responses, which suppress cancer progression [15]. Moreover, galectin-8 has been shown to regulate cell–matrix interactions in vitro [16]. Namely, secreted galectin-8 may function as a physiological modulator of cell adhesion by interacting with integrins, which can either increase or decrease cell adhesion, depending on extracellular factors [17]. It was shown in several cell types that secreted galectin-8 inhibited cell adhesion; however, immobilized galectin-8 had the potential to stimulate cellular attachment, spreading and migration [16]. Kolundžić et al. showed that galectin-8 is strongly expressed in the villous and extravillous trophoblasts of the cell column, as well as decidual stromal cells during early pregnancy [18], suggesting its potential role in the placentation process. Although the expression of galectin-8 has been reported at the fetal–maternal interface [18], its physiological role during pregnancy is theoretical, based on functional studies in different settings [19]. There is a limited understanding of galectin-8’s functions in trophoblasts. Based on the localization of galectin-8 in the placenta in the first trimester of pregnancy and its function in other cell types as described, we investigated the role of galectin-8 in EVT cells.

## 2. Results

### 2.1. Effects of Galectin-8 on Cell Viability and Cell Cycle Distribution

**Figure 1:**
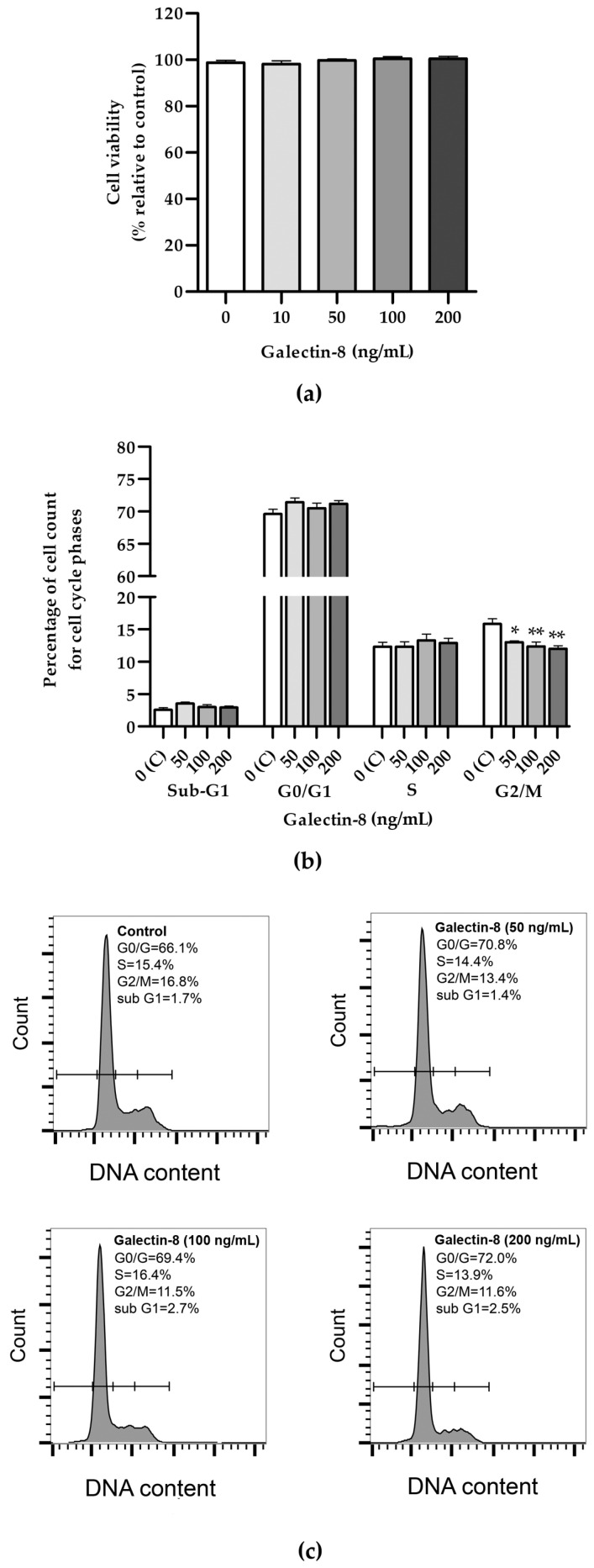
(**a**) The effects of rh galectin-8 on HTR-8/SVneo cell viability. Results are presented as percentages of untreated control values (mean + SEM). The experiment was performed four times with six replicates each. (**b**) The effects of rh galectin-8 (50, 100 and 200 ng/mL) on the HTR-8/SVneo cell cycle. Results are presented as the percentages of cells in the sub-G1, G0/G1, S, G2/M phases of the cell cycle. The experiments were performed three times in three replicates. (**c**) Representative graphs of cell cycle analysis showing percentages of various phases of the cell cycle for the control and each galectin-8 treatment group (50, 100 and 200 ng/mL). * *p* < 0.05 and ** *p* < 0.01.

An MTT assay was used to assess the effects of galectin-8 on HTR-8/SVneo cell viability. The results showed that rh galectin-8, at the concentrations used (10, 50, 100 and 200 ng/mL), did not affect the viability of HTR-8/SVneo cells after 24 h incubation, compared to the non-treated control (Figure 1a).

Further, the effect of rh galectin-8 on cell cycle distribution was examined (Figure 1b). No difference between the groups could be seen for the Sub-G1, G0/G1 and S phases for the same concentrations. A negative effect was observed for all tested concentrations of rh galectin-8 (50, 100 and 200 ng/mL) at the G2/M phase. There was a reduction in cells from 15.8% in the control group to 13.0% (*p* < 0.05), 12.4% (*p* < 0.01) and 12.0% (*p* < 0.01) as seen at 50, 100 and 200 ng/mL rh galectin-8, respectively.

### 2.2. Determination of rh Galectin-8 Genotoxicity in HTR-8/SVneo Cells

**Figure 2:**
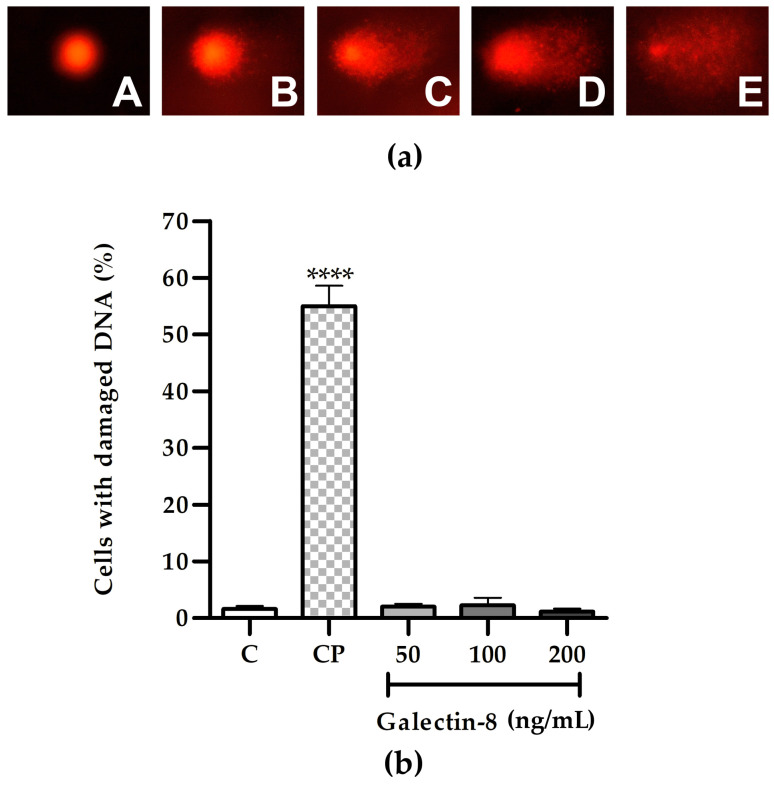
(**a**) Representative images of comet assay categories: A—no DNA damage, B—low, C—medium, D—high and E—total DNA damage. (**b**) The effect of exogenously added rh galectin-8 on the DNA damage of HTR-8/SVneo cells, as determined using the comet assay. Results are presented as the percentage of cells with damaged DNA (mean + SEM). C = control, CP = control with hydrogen peroxide (H_2_O_2_). The experiments were performed three times with two replicates each. **** *p* < 0.0001.

Following the 24 h incubation of HTR-8/SVneo cells with rh galectin-8 at the concentrations used (50, 100 and 200 ng/mL), the results of the comet assay showed the absence of genotoxic effects, as shown in Figure 2b. There was no change in the percentage of cells with DNA damage at any of the used concentrations of rh galectin-8 compared to the non-treated control. Classes of comets representative of the degree of DNA damage are presented in Figure 2a.

### 2.3. Rh Galectin-8 Affects Cell Adhesion of HTR-8/SVneo Cells on Matrigel and Collagen-Coated Surfaces

The adhesion of HTR-8/SVneo cells was studied using plastic 96-well plates, either uncoated or coated with Matrigel or collagen type I, with rh galectin-8-treated cells (10, 50, 100 and 200 ng/mL).

**Figure 3:**
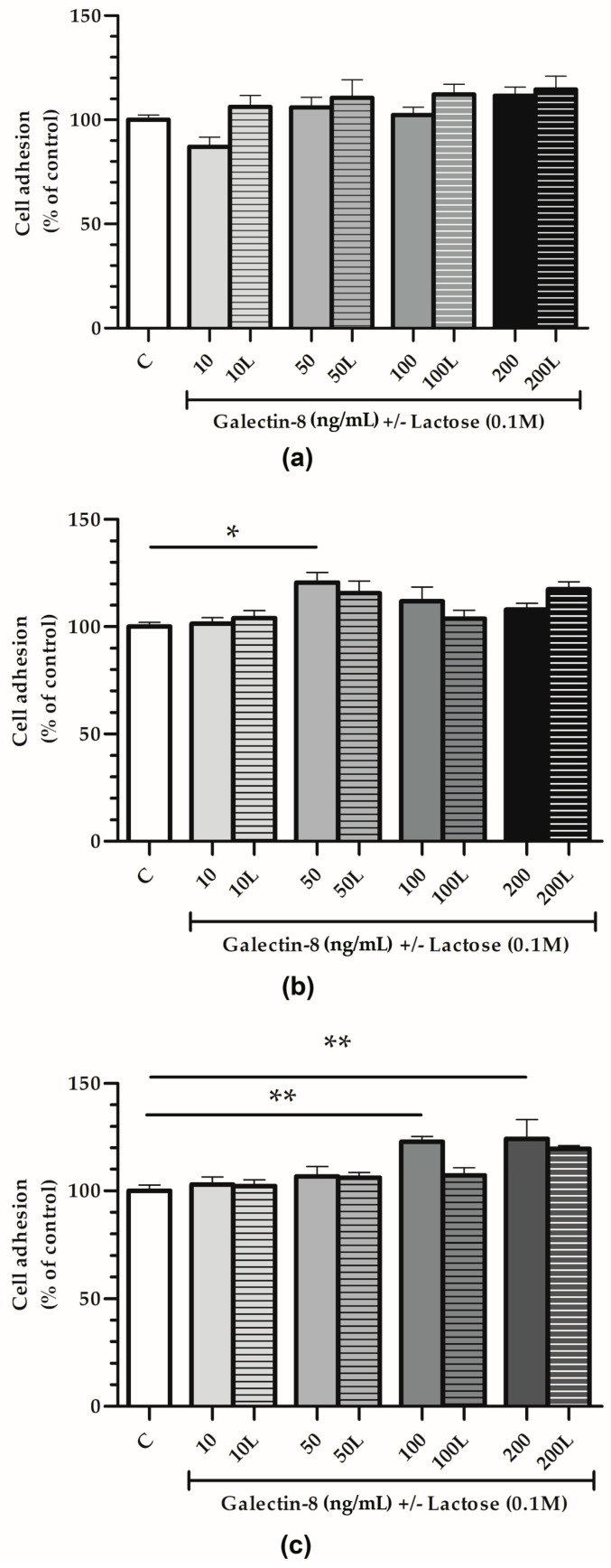
The effect of exogenously added rh galectin-8 on HTR-8/SVneo cell adhesion. Adhesion was examined on (**a**) plastic, (**b**) Matrigel and (**c**) collagen type I-coated surfaces after 2 h incubation at 37 °C at 5% CO_2_. The experiment was performed three times in triplicate. Results are presented as percentages of untreated control values = C (mean + SEM). L = lactose (0.1 M). * *p* < 0.05 and ** *p* < 0.01.

For the plastic surfaces (Figure 3a), no significant effect of rh galectin-8 (10, 50, 100 and 200 ng/mL) on HTR-8/SVneo cell adhesion was observed.

On the other hand, rh galectin-8 stimulated cell adhesion on Matrigel-coated surfaces (Figure 3b) by 120.6% at 50 ng/mL (*p* < 0.05). When lactose was added together with rh galectin-8, this stimulatory effect was absent, i.e., no difference between the control and treatment (rh galectin-8 + lactose) was observed.

Finally, for the collagen-coated surfaces (Figure 3c), rh galectin-8 stimulated the adhesion of HTR-8/SVneo cells by 122.8% at 100 ng/mL and 124.2% at 200 ng/mL with a (*p* < 0.01 for both concentrations). Moreover, 0.1 M lactose (L) was used to assess the inhibition of rh galectin-8 through lectin-type interactions. Similar to the cell adhesion to Matrigel, this stimulatory effect was lost in the presence of lactose.

### 2.4. Increased Galectin-8 Availability Promotes Migration and Invasion of HTR-8/SVneo Cells and Primary Cytotrophoblasts

**Figure 4:**
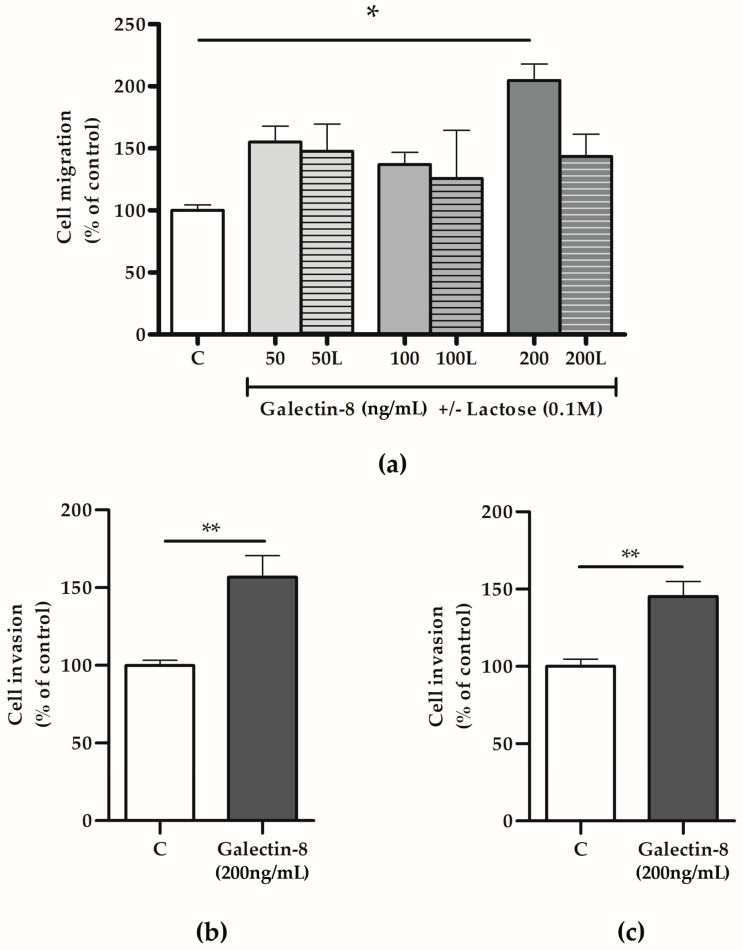
The effects of rh galectin-8 with and without lactose on (**a**) HTR-8/SVneo cell migration. Experiments were performed three times in duplicate. The effects of exogenously added rh galectin-8 on (**b**) HTR-8/SVneo and (**c**) primary cytotrophoblast cell invasion. C = control, L = lactose (0.1 M). Results are presented as percentages of untreated control values (mean + SEM). The experiments were repeated three times in duplicate. * *p* < 0.05, ** *p* < 0.01.

To test the hypothesis that galectin-8 could act as a paracrine regulator of the trophoblast invasion process, HTR-8/SVneo cells were treated with rh galectin-8 and its effects on trophoblast migration and invasion assessed in vitro. The treatment stimulated HTR-8/SVneo cell migration, as shown in Figure 4a. Significant stimulatory action of rh galectin-8 on cell migration was observed at 200 ng/mL, which was 204.8% (*p* < 0.05) compared to the untreated control. The addition of lactose neutralized the stimulatory effect of rh galectin-8 at 200 ng/mL.

Considering that rh galectin-8 at a concentration of 200 ng/mL exhibited significant effects on HTR-8/SVneo cell migration, next, we applied the same concentration to assess galectin-8’s effects on the trophoblast invasion capacity. Here, rh galectin-8 was shown to stimulate HTR-8/SVneo cell invasion at 200 ng/mL by 156.7% (*p* < 0.01), as shown in Figure 4b, and isolated primary cytotrophoblasts by 145.3% (*p* < 0.01) compared to the control, as shown in Figure 4c.

### 2.5. Galectin-8 Does Not Affect Expression of Integrin Subunits Alpha-1 and Beta-1

The effects of rh galectin-8 on integrin subunits alpha-1 and beta-1 were studied at the protein and mRNA level. These subunits comprise the receptor for laminin/collagen and are indispensable for the trophoblast invasion process.

**Figure 5:**
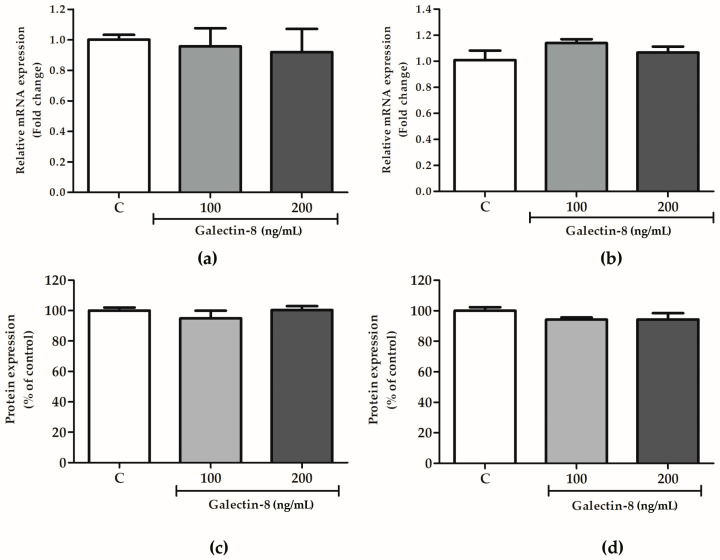
The effects of rh galectin-8 on integrin subunits (**a**) alpha-1 and (**b**) beta-1 at the mRNA level. The results are presented as relative mRNA expression levels from three different experiments in duplicate (mean + SEM). C = control. The effects of exogenously added rh galectin-8 on HTR-8/SVneo integrin (**c**) alpha-1 and (**d**) beta-1 subunit protein expression using a cell-based ELISA (cELISA) assay. Results are presented as percentages of untreated control values (mean + SEM). The experiment was performed 4 times with at least four replicates each. C = control.

The effect of rh galectin-8 on integrins alpha-1 and beta-1 was analyzed at the mRNA level. Integrin alpha-1 and beta-1 subunit expression at the mRNA level was not changed in HTR-8/SVneo cells following rh galectin-8 treatment (100 and 200 ng/mL) for 24 h compared to the control cells (Figure 5a,b). In addition, for the assessment of these molecules at the protein level, the cell-based ELISA assay was used, as described in the Section 4. No effect of rh galectin-8 at concentrations of 100 and 200 ng/mL was observed on HTR8/SVneo cells’ expression of integrin subunits alpha-1 and beta-1 after 24 h incubation compared to the control value from untreated cells, as shown in Figure 5c,d.

### 2.6. Rh Galectin-8 Affects MMP-2 and MMP-9 Levels

To further decipher the mechanism behind the galectin-8-mediated stimulation of trophoblast invasion, the effect of exogenously added rh galectin-8 on MMP-2 and MMP-9 was studied at the protein and mRNA level.

**Figure 6:**
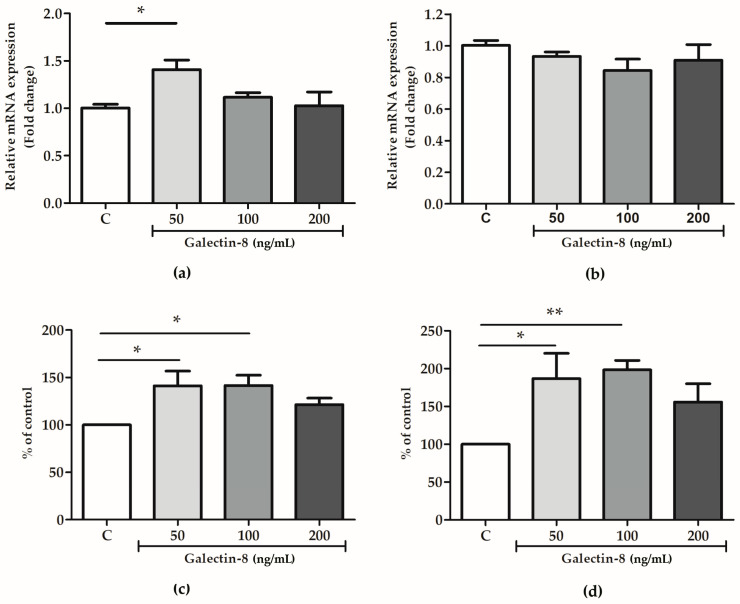
The effects of rh galectin-8 on (**a**) MMP-2 and (**b**) MMP-9 relative mRNA expression. The results are presented as a fold change in mRNA expression from three different experiments in duplicate (mean + SEM). C = control. The effects of exogenously added rh galectin-8 on (**c**) MMP-2 and (**d**) MMP-9 levels. Results are presented as percentages of untreated cell control values (mean + SEM). C = control. The experiments were performed at least three times in duplicate. * *p* < 0.05, ** *p* < 0.01.

As shown in Figure 6a, the rh galectin-8 treatment at 50 ng/mL induced the maximum change in MMP-2 mRNA expression, which was a 1.408-fold change (*p* < 0.05). However, no significant change in the mRNA level for MMP-9 was observed for the tested concentrations of rh galectin-8 (Figure 6b). Meanwhile, rh galectin-8 increased the MMP-2 protein levels, as shown in Figure 6c, to 141.2% (*p* < 0.05) and 141.6% (*p* < 0.05) at the rh galectin-8 concentrations of 50 and 100 ng/mL, respectively, and for MMP-9 to 186.8% (*p* < 0.05) and 198.5% (*p* < 0.01) at the rh galectin-8 concentrations of 50 ng/mL and 100 ng/mL, respectively (Figure 6d). A representative zymogram is shown in Supplementary Figure S1.

## 3. Discussion

To the best of our knowledge, the role of galectin-8 in EVT function has not been studied before. Thus far, galectin-8’s function has only been presumed based on previous studies reporting its localization in trophoblasts and its function in other cell types. This study aims to extend the findings on galectin-8’s localization in EVTs [18]. The significance of galectin-8 for EVT function, such as adhesion, migration and invasion, as well as the expression of trophoblast invasive phenotype markers, namely integrins and MMPs, was studied.

After an extensive literature search, no data regarding the tissue levels of galectin-8 could be found. However, information on plasma or serum concentrations has been documented. In one study, the mean plasma galectin-8 level from healthy controls was 0.25 ng/mL [20]. In another study, the mean serum galectin-8 level in healthy controls was 19.4 ng/mL [21]. Taking into account that these concentrations are for plasma/serum samples, we hypothesized that the galectin-8 concentrations in tissue would be higher where the galectin signaling is primarily autocrine or paracrine.

Using the MTT assay, rh galectin-8 was shown not to affect the viability of HTR-8/SVneo cells and exhibited no genotoxicity in the comet assay. Further, a tendency to increase was noted regarding the frequency of cells treated with rh galectin-8 in the G0/G1 and S phases of the cell cycle, although there was no difference between the groups. This finding is consistent with previous evidence that soluble galectin-8 inhibits cellular growth in the H1299 cell line (human non-small-cell lung carcinoma cells), by promoting the accumulation of the cyclin-dependent kinase inhibitor p21. p21 accumulation was mediated, at least partially, through the activation of c-Jun N-terminal kinases (JNK) and protein kinase B (PKB), whose phosphorylation was markedly increased in galectin-8-treated cells [22]. p21, as with its other related counterparts, p27 and p57, blocks progression through the cell cycle at the G1/S and G2/M checkpoints by forming ternary complexes with cyclin-dependent kinases, thus inhibiting their enzymatic activity [23]. The increase in the frequency of cells in the G0/G1 and S phases was accompanied by a mild but statistically significant reduction in the frequency of cells distributed in the G2/M phase of the cell cycle, for all tested concentrations of rh galectin-8 treatment. However, in another study, galectin-8 was found to promote keratinocyte proliferation. When galectin-8 was upregulated, cell proliferation was increased, which resulted in epidermal hyperplasia in psoriasis [24]. The differences in the effects of galectin-8 may be due to the fact that, in our study, galectin-8 was added exogenously, as opposed to increasing its endogenous expression. In terms of the genotoxicity and cell cycle influence of galectins, galectin-3 was previously found to be involved in the cellular response to DNA damage and the inhibition of cell cycle progression. Namely, in *LGALS3*-silenced HEK293FT human cells exposed to different DNA damage agents, there was delayed DNA damage repair (DDR) response activation and reduced G2/M cell cycle arrest, which allowed the cells to undergo mitosis independently of DNA errors [25]. In glioma cells, the silencing of *LGALS1* and *LGALS3* resulted in decreased resistance to radiotherapy, while DNA damage induced by radiation was increased [26]. In most of the studies on breast cancer and galectins, galectin-3 expression played a protective role in cell survival through various pathways, including the response to DNA damage and repair, as well as the inhibition of apoptosis following chemotherapy treatment [27]. Thus, it is plausible that, similarly to the above-mentioned galectins, excessive galectin-8 could also promote cell survival and reduce DNA damage, which remains to be confirmed.

Other studies have demonstrated that galectin-8 affects the cellular adhesive properties in different cells, where the effect of galectin-8 has been shown to attenuate or stimulate cell adhesion under different experimental conditions [16,28,29]. In this study, the effect of rh galectin-8 on cell adhesion was investigated on three different surfaces: plastic-, collagen type I- and Matrigel-coated surfaces. It is of note that Matrigel has been documented to contain galectin-8, as described by Bojić-Trbojević et al. [12]. A stimulating effect on cell adhesion was observed on Matrigel-coated surfaces at the rh galectin-8 concentration of 50 ng/mL and on collagen-coated surfaces at the rh galectin-8 concentrations of 100 ng/mL and 200 ng/mL, where lactose partially inhibited its effect, indicating that galectin-8’s influence is realized partially by lectin activity. However, no significant effect of rh galectin-8 on HTR-8/SVneo cells was observed on plastic (uncoated) surfaces. The results showed that galectin-8 is involved in the complex process of EVT cell adhesion in vitro and that its influence is realized partially by lectin activity, suggesting its role in regulating cell adhesion in early pregnancy. The dual effect of added or immobilized galectin-8 was also shown on several cell types [16]. Immobilized galectin-8 promoted cell adhesion, while added lectin negatively regulated this process. Another study showed that galectin-8 stimulated cell adhesion and the spreading of trabecular meshwork cells [30].

As hypothesized based on its expression in EVTs in the cell column, galectin-8 has been shown to stimulate HTR-8/SVneo cell migration at 200 ng/mL in our study. Galectin-8 has been shown to affect cell migration in other cell types as well. For example, in glioblastoma U87 cells, exogenously added galectin-8 induced directional migration [31]. Additionally, exogenously added rh galectin-8 has also been shown to stimulate human umbilical vein endothelial cells’ migration in vitro, although this was in the presence of vascular endothelial growth factor (VEGF) [32]. Furthermore, in another study, galectin-8 coated on plastic markedly decreased the migration of two types of colon cancer cells, HCT-15 and CoLo-201 [33]. Our results demonstrated that exogenously added rh galectin-8 (200 ng/mL) stimulated the invasion of both HTR-8/SVneo and primary cytotrophoblast cells. This was also evident in other cell types studied. For example, the results from one study suggested that galectin-8 (along with galectin-1 and -3) may be associated with the tumor astrocyte invasion of the brain parenchyma, where it was shown that these galectins stimulated glioblastoma cell migration in vitro [34]. The effects of galectin-8 on trophoblast cell invasion reported here may not be attributed solely to exogenously added proteins but also to secreted trophoblast galectin-8 [35], as well as that present in Matrigel [12].

To begin to elucidate the mechanism behind the galectin-8-mediated impact on trophoblast cell function, the expression of molecular mediators—integrins and MMPs—was evaluated in HTR-8/SVneo cells. Our results suggest that the stimulation of trophoblast cell invasion by added rh galectin-8 is not mediated through the modulation of integrin subunits alpha-1 and beta-1. This does not exclude the involvement of other adhesion molecules, which needs to be explored further. Our previous results have shown that two other galectin family members, galectin-1 and -3, also participate in the trophoblast invasion process [11,12]. The effects of all three galectins were at least in part exerted through lectin interactions, as evidenced when adding inhibitory sugar lactose to the system. Here, rh galectin-8 (50 ng/mL) stimulated the expression of MMP-2, while the MMP-9 mRNA levels were not affected by the rh galectin-8 treatment of HTR-8/SVneo cells. In terms of protein levels, both MMP-2 and MMP-9 in the conditioned media of treated HTR-8/SVneo cells were shown to be increased by galectin-8 (at 50 and 100 ng/mL for both MMPs). MMP-2 and MMP-9 are crucial enzymes with a central role in endometrial tissue remodeling, including the remodeling of the uterine vasculature during pregnancy, by facilitating the degradation of ECM components and contributing to cytotrophoblast invasion [36]. The increase in these enzymes’ activity indicates that galectin-8 has a role in regulating tissue remodeling in the placenta. Data in the literature relating galectin-8 to MMP regulation are scarce. Galectin-8 has been shown to increase the MMP-3-mediated processing of proMMP-9 in human neutrophils [37].

This study found that exogenously added rh galectin-8 stimulated the EVTHTR-8/SVneo cell line in terms of adhesion, migration and invasion, as well as stimulating the cell invasion of primary cytotrophoblasts in vitro. These processes were partially mediated by lectin-type interactions, as they were, to some extent, inhibited by the addition of lactose. Galectin-8 also positively affected the levels of secreted MMP-2 and MMP-9, as well as MMP-2 expression at the mRNA level. Taken together with the confirmed presence of galectin-8 in EVTs [18], it can be concluded that galectin-8 may act as an important local paracrine mediator of the trophoblast invasion process, together with galectin-1 and galectin-3.

## 4. Materials and Methods

### 4.1. Cell Culture

The RPMI 1640 medium, antibiotic/antimycotic solution and fetal calf serum (FCS) were acquired from Gibco (Paisley, UK). The HTR-8/SVneo cell line—an EVT cell line—was kindly provided by Dr. Charles H Graham (Queen’s University, Kingston, ON, Canada). This cell line was created by increasing the lifespan of human first-trimester trophoblasts using simian virus 40 large T antigen [38]. The cells were cultured in RPMI 1640 medium with 10% FCS (*v/v*) and 1% antibiotic/antimycotic solution (*v/v*) (complete medium).

### 4.2. Cytotrophoblast Isolation Protocol

Placental tissue was acquired from subjects who had undergone the voluntary termination of pregnancy at the Clinic for Obstetrics and Gynecology, Clinical Center of Serbia, Belgrade, after verbal consent was given and with the approval of the institutional Ethics Committee (approval no. 02-1048/3). Cytotrophoblast cells were isolated from placentas (6–12 weeks) using trypsin digestion, as previously described [39], with some modifications [11,12].

### 4.3. Recombinant Human Galectin-8

The recombinant human (rh) galectin-8 was acquired from R&D Systems (Minneapolis, MN, USA). Galectin-8 was diluted in serum-free RPMI 1640 medium with 0.1% bovine serum albumin (BSA). The rh galectin-8 concentrations were selected based on our previous studies with galectin-1 and galectin-3.

### 4.4. MTT—Cell Viability Assay

HTR-8/SVneo cells were seeded (2 × 10^4^ cells/well) into 96-well plates and grown in 100 μL complete medium for 24 h. After a washing step with PBS, the HTR-8/SVneo cells were incubated for 24 h in treatment media containing different concentrations of rh galectin-8 (10, 50, 100 and 200 ng/mL) in 100 μL 0.1% BSA in RPMI 1640 medium, including a control that had no added galectin-8. The medium was removed, the well plate was washed once with PBS, and 100 μL of MTT solution (Sigma-Aldrich, Burlington, CA, USA) in PBS at a concentration of 0.5 mg/mL was added, and the sample was incubated for 2 h at 37 °C. To dissolve the formazan crystals, 10% SDS with 0.01 N HCl was added (100 μL/well), and the sample was incubated for 24 h at 37 °C. The absorbance was read at 570 nm (Epoch, BioTek, Winooski, VT, USA). The experiment was repeated three times with six replicates for each treatment.

### 4.5. Comet Assay

HTR-8/SVneo cells were seeded (2 × 10^4^ cells/well) into 96-well plates and grown in 100 μL complete RPMI 1640 medium for 24 h. After a washing step with PBS, the HTR-8/SVneo cells were incubated for 24 h in treatment media containing different concentrations of rh galectin-8 (50, 100 and 200 ng/mL) in 100 μL 0.1% BSA in serum-free RPMI medium, including a control that had no added galectin-8. For the positive control, hydrogen peroxide (H_2_O_2_) was used, where the cells were incubated with 30 µM H_2_O_2_ in serum-free RPMI medium for 30 min at 37 °C. The HTR-8/SVneo cells were detached from the plates using trypsin–EDTA solution and resuspended in 100 μL complete RPMI 1640 medium. The comet assay was performed as described by Bruić et al. [40], with some modifications. Briefly, 80 μL of a HTR-8/SVneo suspension was mixed with 120 μL of low-melting-point agarose (Sigma-Aldrich, St. Louis, MO, USA) and pipetted onto glass microscope slides precoated with 1% normal-melting-point agarose (Sigma-Aldrich, St. Louis, MO, USA), spread by placing the coverslip on top and kept at 4 °C for 10 min to solidify. The coverslips were removed and the slides were placed in cold lysing solution (2.5 M NaCl, 100 mM EDTA, 10 mM Tris, 1% Triton X100 and 10% dimethylsulfoxide, pH 10 adjusted with NaOH) at 4 °C overnight. Subsequently, the slides were placed in cold electrophoresis solution for 30 min (300 mM NaOH, 1 mM EDTA) and electrophoresis was run at 25 V and 300 mA for 30 min. The slides were rinsed with PBS three times and stained with ethidium bromide (20 µg/mL). The comets were analyzed 15 min after staining at 100× magnification on an Olympus IX 73 microscope (Olympus Optical Co., GmbH, Hamburg, Germany). The DNA damage assessment was performed according to Anderson et al. [41]. The DNA damage of HTR/SVneo cells was visually determined by the quantification of the amount of DNA that had migrated from the nucleus, which was projected as a ‘tail’. The DNA tails were visually assessed by an experienced analyst and were classified into five categories depending on the level of DNA damage: (A) no damage, <5%; (B) low level of damage, 5–20%; (C) medium level of damage, 20–40%; (D) high level of damage, 40–95%; (E) total damage, >95%. The analysis was performed on 100 cells from each of 2 replicate slides. DNA damage was designated as DNA migration over 5% (sum of B + C + D + E comet classes) and values were reported as the percentage of cells with DNA damage.

### 4.6. Cell Cycle—Flow Cytometry

HTR-8/SVneo cells were seeded (2 × 10^5^ cells/well) into 24-well plates and grown in 300 μL RPMI 1640 medium containing 10% FCS with 1% antibiotic/antimycotic mixture for 24 h. The cells were washed x2 with PBS; 300 μL of RPMI medium was added and they were incubated for 24 h at 37 °C. Galectin-8 treatments (50, 100 and 200 ng/mL) were added, and a control cell sample without galectin-8 in 0.1% BSA/RPMI medium only was also included. The cells were incubated for a further 24 h at 37 °C.

The HTR-8/SVneo cells were detached from the plates using trypsin–EDTA solution and washed x2 in PBS. The cells were resuspended in 200 μL of PBS, and, with constant vortexing, 3 mL of ice-cold 70% ethanol was slowly added. The cells were fixed for 1 h at 4 °C and washed twice in PBS afterwards. Finally, 500 μL of propidium iodide solution was added and the cell distribution across the cell cycle phases was analyzed using the BD^®^ LSR II Flow Cytometer (Becton Dickinson, Mountain View, CA, USA).

### 4.7. Cell Adhesion

Cell adhesion assays were performed on plastic/uncoated wells and wells precoated with collagen type I (BD Biosciences, Bedford, MA, USA) and Matrigel (BD Biosciences, Bedford, MA, USA) at 250 ng/well for 1 h at 37 °C. The plates were subsequently blocked for non-specific binding using 1% BSA in PBS for 30 min at 37 °C and washed with PBS. The HTR-8/SVneo cells were detached with 0.05% trypsin/0.02% EDTA in PBS and resuspended in 0.1% BSA-RPMI 1640 medium at a final concentration of 2.5 × 10^5^ cells/mL. The cells were preincubated for 1 h at 37 °C with different concentrations of rh galectin-8 (10, 50, 100 and 200 ng/mL) with or without 0.1 M lactose, including a control that had no added rh galectin-8, with occasional agitation. The cells were subsequently plated at 2.5 × 10^4^ cells per well in 100 µL medium. After incubation for 2 h at 37 °C, the unattached cells were removed, and the wells were rinsed once with PBS and fixed with ice-cold acetone/methanol (1:1) for 10 min. The attached cells were stained by adding 0.05% crystal violet in 25% methanol at 50 µL/well for 5 min. The excess dye was removed by immersing the plates at least three times in dH_2_O and drying them at RT. The incorporated crystal violet stain was dissolved in 0.1 M sodium citrate in 50% ethanol at 100 µL/well and the light absorbance was read at 570 nm (Epoch, BioTek, Winooski, VT, USA). The stain intensity was proportional to the number of attached cells. The experiments were repeated at least three times with three replicates each.

### 4.8. Cell Migration

The effects of various concentrations of rh galectin-8 (50, 100 and 200 ng/mL) on HTR-8/SVneo cell migration were investigated using the ‘wound healing’ assay. HTR-8/SVneo cells were first seeded at 3 × 10^5^ cells/well in 400 μL of complete medium in a 24-well plate for 24 h or until confluent, at which point a gap between the cells was created across the middle of the well by scraping a straight line across the center of the well using a sterile 200 µL pipette tip. After rinsing with PBS, RPMI 1640 containing 0.1% BSA with or without rh galectin-8 was added with or without 0.1 M lactose. The pre-selected fields were photographed at the zero point when the scratch was created and after 24 h. The distances crossed by the cells after 24 h were measured using an electronic ruler in ImageJ 1.53t. The data are presented as the percentage of migration of treated cells compared to the control mean, which was set as 100%. The experiment was repeated three times in duplicate.

### 4.9. Cell Invasion

Transwell Matrigel invasion assays were performed as previously described [11]. HTR-8/SVneo or primary cytotrophoblast cells were seeded in transwell inserts (8 µm pore size; MilliporeSigma, Burlington, MA, USA) and placed in the wells of a 24-well plate. The inserts were coated with 10 μL of growth factor reduced Matrigel diluted in serum-free RPMI 1640 (2.5 mg/mL). Cells were placed in the upper well at 1 × 10^5^ in 200 µL of RPMI for HTR-8/SVneo cells or 0.1% BSA DMEM/F12 medium for CT, without (C) or with rh galectin-8 (200 ng/mL), and 500 µL of the identical medium corresponding to the upper well was applied to the lower well. The cell invasion assay was run for 24 h at 37 °C. Cells were stained using crystal violet and counted on the underside of the filter and the pores occupied by cells in sequential fields of the whole insert. The data presented are expressed as percentages of the mean of the control value, which was set at 100%. The experiment was repeated at least three times in duplicate.

### 4.10. qPCR

HTR-8/SVneo cells were first seeded at 2.5 × 10^5^ cells/well in 300 μL of 10% FCS RPMI 1640 medium in a 24-well plate for 24 h. After a washing step with PBS, the HTR-8/SVneo cells were incubated for 24 h in treatment media containing different concentrations of rh galectin-8 in duplicate (50, 100 and 200 ng/mL) in 300 μL of 0.1% BSA RPMI 1640 medium, including a control that had no added galectin-8.

Total RNA was isolated from the treated HTR-8/SVneo cells using TRIzol (Invitrogen, Carlsbad, CA, USA) according to the manufacturer’s instructions. First-strand cDNA was synthesized from 1 μg of total RNA, using 0.5 μg of Oligo(dT) 12–18 primers (Invitrogen, Carlsbad, CA, USA), 250 μM of each dNTP and 200 U of RevertAid reverse transcriptase (Fermentas, Vilnius, Lithuania). Real-time PCR was performed using the 7500 Real-Time PCR System (Applied Biosystems, Carlsbad, CA, USA). The reaction mixture contained 1 μL of cDNA, 5 μL 2x SYBR^®^ Green PCR Master Mix (Applied Biosystems, Carlsbad, CA, USA) and specific forward and reverse primers in a final concentration of 0.5 μM. Reactions were run at 95 °C for 10 min, followed by 40 cycles of 15 sec at 95 °C and 1 min at 60 °C. Melting curve analysis was performed to verify the amplification specificity. The expression levels of *ITGA1*, *ITGB1*, *MMP2* and *MMP9* were normalized to that of the housekeeping gene *GAPDH*. Calculations were performed using the 2^−ddCt^ method.

The sequences of the specific primers were as follows.
**Gene****Primer Sequence***ITGA1*Forward: 5′-GGTTCCTACTTTGGCAGTATT-3′
Reverse: 5′-AACCTTGTCTGATTGAGAGCA-3′*ITGB1*Forward:5′-GTGGTTGCTGGAATTGTTCTTATT-3′
Reverse:5′-TTTTCCCTCATACTTCGGATTGAC-3′*MMP2*Forward:5′-TGCGACCACAGCCAACTACG-3′
Reverse:5′-ACAGACGGAAGTTCTTGGTGTAGG-3′*MMP9*Forward:5′-TGACAGCGACAAGAAGTG-3′
Reverse:5′-CAGTGAAGCGGTACATAGG-3′*GAPDH*Forward:5′-GAAGGTGAAGGTCGGAGT-3′
Reverse:5′-GAAGATGGTGATGGGATTTC-3′

### 4.11. Cell-Based ELISA Assay (cELISA)

HTR-8/SVneo cells were seeded (2 × 10^4^ cells/well) into 96-well plates and grown in 100 μL of complete medium for 24 h. After a washing step with PBS, the HTR-8/SVneo cells were incubated for 24 h in treatment media containing different concentrations of rh galectin-8 (100 and 200 ng/mL) in 100 μL 0.1% BSA in RPMI 1640 medium, including a control that had no added galectin-8. The cells were washed twice with PBS and fixed with a freshly prepared, cold acetone/methanol solution for 10 min. The plates were air-dried and then kept at 4 °C with a moisture absorbent until the antibody detection procedure was carried out.

A total of 200 μL of PBS was added to each well for the cells to rehydrate and then discarded. Then, 100 μL of hydrogen peroxide (0.3% in PBS) was added to each well and they were incubated for 30 min in the dark to block endogenous peroxidase activity. After a washing step with PBS, the cells were blocked with 100 μL of 1% BSA in PBS for 30 min and discarded. Mouse anti-integrin alpha-1 Ab (R&D Systems, Minneapolis, MN, USA) diluted 1:400 with 1% BSA in PBS and rabbit anti-integrin beta-1 Ab (Millipore Sigma, Burlington, MA, USA) diluted 1:500 with 1% BSA in PBS were added to each well (50 μL/well) and they were left in a humid container overnight at 4 °C.

The wells were washed five times with PBS and incubated for 1 h with HRP-linked anti-mouse Ab (Cell Signaling Technology, Danvers, MA, USA) or HRP-linked anti-rabbit Ab (Cell Signaling Technology, Danvers, MA, USA) diluted 1:2000 with 1% BSA in PBS. The wells were washed x5 with PBS and a substrate solution was added until a color developed. The reaction was terminated by adding the stop solution and the absorbance was read at 450 nm (Epoch, BioTek, Winooski, VT, USA).

### 4.12. Zymography (SDS-PAGE Gelatin Zymography)

Matrix metalloproteinase (MMP-2 and MMP-9) gelatinolytic activity was studied semi-quantitatively in the conditioned media of treated HTR-8/SVneo cells. The cells were first seeded at 3 × 10^5^ cells/well in 300 μL of complete medium in a 24-well plate for 24 h. After a washing step with PBS, the HTR-8/SVneo cells were incubated for 24 h at 37 °C in treatment media containing different concentrations of rh galectin-8 in duplicate (50, 100 and 200 ng/mL) in 300 μL RPMI 1640 medium with 0.1% BSA, including a control that had no added galectin-8. The gelatinase activity was determined in the centrifuged media of HTR-8/SVneo cells using SDS-PAGE gelatin zymography, where a 7.5% acrylamide gel containing 0.8 mg/mL gelatin under non-reducing conditions was used. The experiments were repeated three times with the proteins loaded per lane at the same concentration for all samples. The gels were photographed using ChemiDoc MP Imager (Bio-Rad, Hercules, CA, USA) and the bands were measured by densitometric analysis using the ImageMaster TotalLab v2.01 program (Amersham Biosciences, Piscataway, NJ, USA).

### 4.13. Statistical Analysis

The data were analyzed using the GraphPad Prism 10 Demo Software (GraphPad Software, Inc., La Jolla, CA, USA). One-way analysis of variance (ANOVA) with Tukey’s post hoc test (α = 0.05) was used for statistical analyses, since the data passed the normality test. For the invasion assay, an unpaired t-test was used for the analysis of the data. Values were considered significantly different when *p* < 0.05.

## Data Availability

Data available upon request.

## Supplementary Materials

The following supporting information can be downloaded at: https://www.mdpi.com/article/10.3390/ijms251810096/s1.
