# Supplementary figures and images for: Galectin-8 Contributes to Human Trophoblast Cell Invasion
Source: Int J Mol Sci. 2024 Sep 20;25(18):10096. doi: 10.3390/ijms251810096 (PMC11431856; doi:10.3390/ijms251810096)

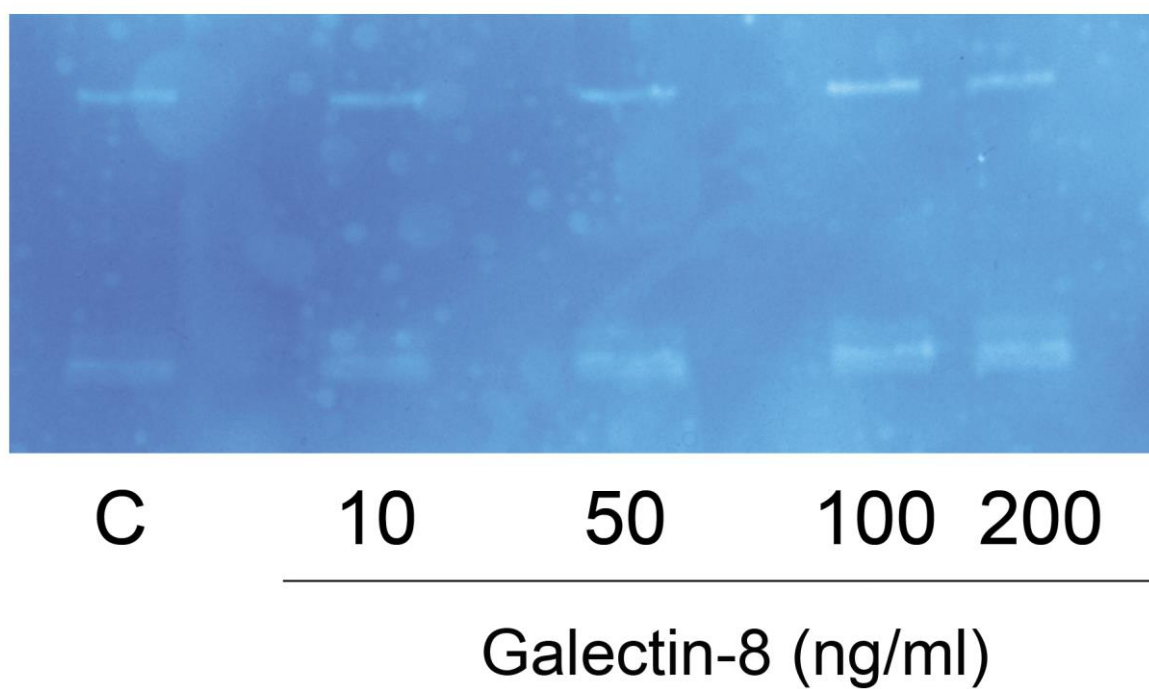

**Figure S1.** Representative zymogram of MMP-2 and MMP-9 in the culture media of HTR-8/SVneo cells.

Supplement: Supplementary file 1 [file ijms-25-10096-s001.zip › ijms-3163320-supplementary.pdf]
